# Supplementary material for: HIV-1 Vpr combats the PU.1-driven antiviral response in primary human macrophages
Source: Nat Commun. 2024 Jun 29;15:5514. doi: 10.1038/s41467-024-49635-w (PMC11217462; doi:10.1038/s41467-024-49635-w)
Supplement: Supplementary file 1 — Supplementary Information [file 41467_2024_49635_MOESM1_ESM.pdf]

**Population**	**Virus-treated**	***gag+/tat+***	***gag-/tat-***
Cluster 0	47880	14641	33239
Cluster 1	1539	214	1325
WT-vpr	13639	6156	7483
Vpr-null	35780	8699	27081

Supplementary Table 1. MDM infection status by population measured with Gag. The number of MDMs treated with the indicated virus (89.6^{wt} or $89.6^{\Delta vpr}$) included in our scRNA-seq analysis (Figure 1E) are indicated. Cells are listed as either virus-treated, *gag+/tat+* or *gag-/tat-* within each cluster, or within infection exposure (WT = 89.6^{wt} and ∆Vpr = $89.6^{\Delta vpr}$) across all three donors.

			Percent Infected	
Donor	Experiment Type	Molecule	89.6 WT vpr	89.6 vpr-null
1	Flow Cytometry	Protein	65%	50%
1	scRNA-seq	mRNA	71%	48%
2	Flow Cytometry	Protein	45%	31%
2	scRNA-seq	mRNA	53%	28%
3	Flow Cytometry	Protein	52%	35%
3	scRNA-seq	mRNA	N/A	(a) 42%
3	scRNA-seq	mRNA	N/A	(b) 54%

Supplementary Table 2. MDM infection rates measured with Gag. Percent infection of 89.6^{wt} and $89.6^{\Delta vpr}$ infected MDMs from each of three donors was determined by quantifying the percent Gag^{+} cells by either flow cytometry (protein) or gene expression levels from scRNA-seq data (mRNA), as indicated by the experiment type and molecule, over the total number of cells analyzed.

ETS Family Transcription Factors

Motif	TF Name	q-value	# Targets w/ Sequence
	ETV4	0.0000	1270
	ETV1	0.0000	1228
	ETS1	0.0000	987
	Fli1	0.0000	1229
	Elk4	0.0000	1038
	Elf4	0.0000	930
	Elk1	0.0000	1013
	ERG	0.0000	1135
	GABPA	0.0000	995
	ELF1	0.0000	925
	EHF	0.0000	876
	ETS	0.0000	674
	ELF5	0.0000	557
	Etv2	0.0000	768
	EWS	0.0001	327
	PU.1	0.0005	316
	ELF3	0.0011	417
	SpiB	0.0038	180
	EWS:FLI1-fusion	0.0063	534
	ETS:RUNX	0.0082	121
	SPDEF	0.0121	624

Homeobox Family Transcription Factors

Motif	TF Name	q-value	# Targets w/ Sequence
	Hoxa13	0.0043	647
	HOXB13	0.0183	292
	Hoxd13	0.0183	1
	Nkx3.1	0.0255	746
	Hoxa9	0.0355	796

bZIP Family Transcription Factors

Motif	TF Name	q-value	# Targets w/ Sequence
	CRE	0.0000	385
	Atf1	0.0043	434
	CREB5	0.0632	203

NRF Family Transcription Factors

Motif	TF Name	q-value	# Targets w/ Sequence
	NRF1	0.0000	544
	NRF	0.0003	1

CCAAT Family Transcription Factors

Motif	TF Name	q-value	# Targets w/ Sequence
	NFY	0.0000	782

ETS/IRF Family Transcription Factors

Motif	TF Name	q-value	# Targets w/ Sequence
	PU.1-IRF	0.0004	670

IRF Family Transcription Factors

Motif	TF Name	q-value	# Targets w/ Sequence
	IRF8	0.0006	209
	IRF2	0.0049	75
	IRF1	0.0453	68
	T1ISRE	0.0504	8

ZF Transcription Factors

Motif	TF Name	q-value	# Targets w/ Sequence
	Sp5	0.0000	1786
	Sp2	0.0000	2047
	KLF1	0.0000	1607
	Sp1	0.0000	1125
	KLF5	0.0000	1787
	KLF3	0.0000	1089
	YY1	0.0000	203
	KLF6	0.0000	1497
	KLF14	0.0000	2044
	Klf9	0.0001	780
	Maz	0.0012	1644
	Klf4	0.0056	531
	GFY	0.0183	200
	ZNF143|ST	0.0567	275

bHLH Family Transcription Factors

Motif	TF Name	q-value	# Targets w/ Sequence
	E-box	0.0000	212
	Usf2	0.0000	290
	MITF	0.0000	505
	TFE3	0.0000	160
	CLOCK	0.0000	402
	USF1	0.0002	371
	c-Myc	0.0006	495
	bHLHE41	0.0036	888
	bHLHE40	0.0041	280
	BMAL1	0.0043	679
	n-Myc	0.0150	432
	Max	0.0228	347
	NPAS	0.0254	612
	HIF2a	0.0636	246

THAP Family Transcription Factors

Motif	TF Name	q-value	# Targets w/ Sequence
	Ronin	0.0227	185

Supplementary Figure 1. Transcription factors and their predicted binding motifs in downregulated genes in MDMs expressing Vpr. Motifs, transcription factor names, significance (FDR q-value), and number of target genes identified by HOMER analysis of genes downregulated in the presence of Vpr (Figure 1E) from several transcription factor families.

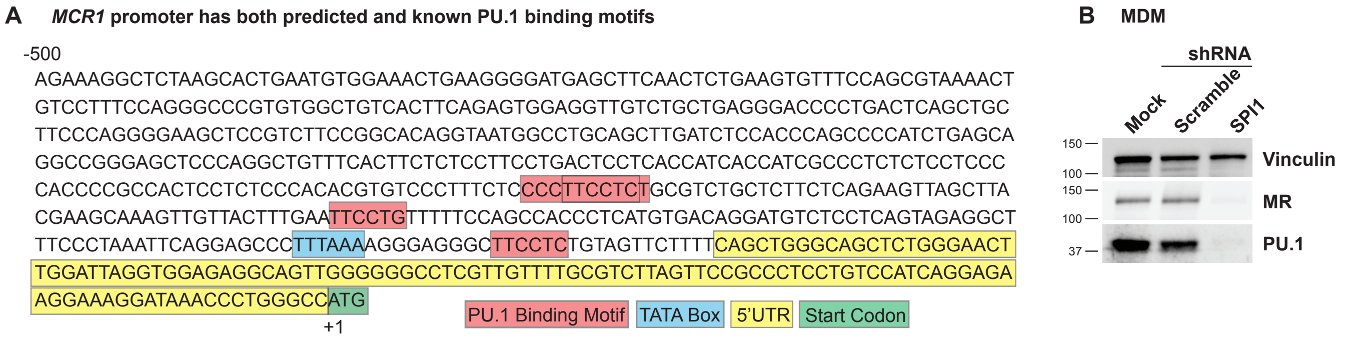

Supplementary Figure 2. The MRC1 promoter contains multiple PU.1 binding motifs. (A) The first 500 bp upstream of the start codon in *MRC1*, the gene that codes for mannose receptor. Previously reported PU.1 binding motifs are outlined in red. The double-box PU.1 motif was identified through inputting the HOMER generated PU.1 motif parameters into FIMO (Find Individual Motif Occurrences). The TATA box is outlined in blue, the 5'UTR in yellow, and the start codon in green. (B) Immunoblot analysis from MDMs stably expressing the indicated shRNAs, n = 2. Source data are provided as a Source Data file.

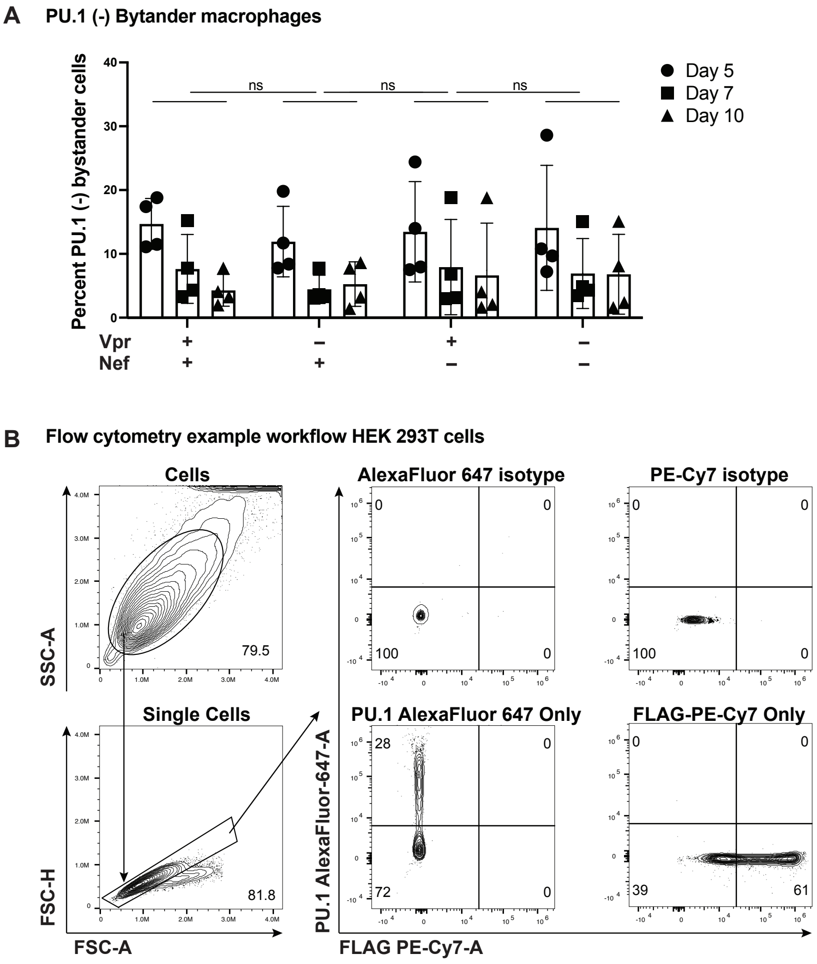

Supplementary Figure 3. PU.1 is not significantly reduced in bystander MDMs at 5-, 7-, and 10-days post infection with replication defective (non-spreading) HIV constructs. (A) Summary graph showing the percentage of infected (GFP^-) cells that do not express PU.1 as determined by flow cytometry as depicted in Figure 4B. The mean +/- standard deviation from n=4 independent donors is shown for each time point. P values were determined using an analysis of variance (ANOVA) with Tukey's multiple comparisons test; ns = not significant. (B) Gating strategy used for flow cytometry in Figures 4 and 5, and Supplementary Figure 4. Source data are provided as a Source Data file.

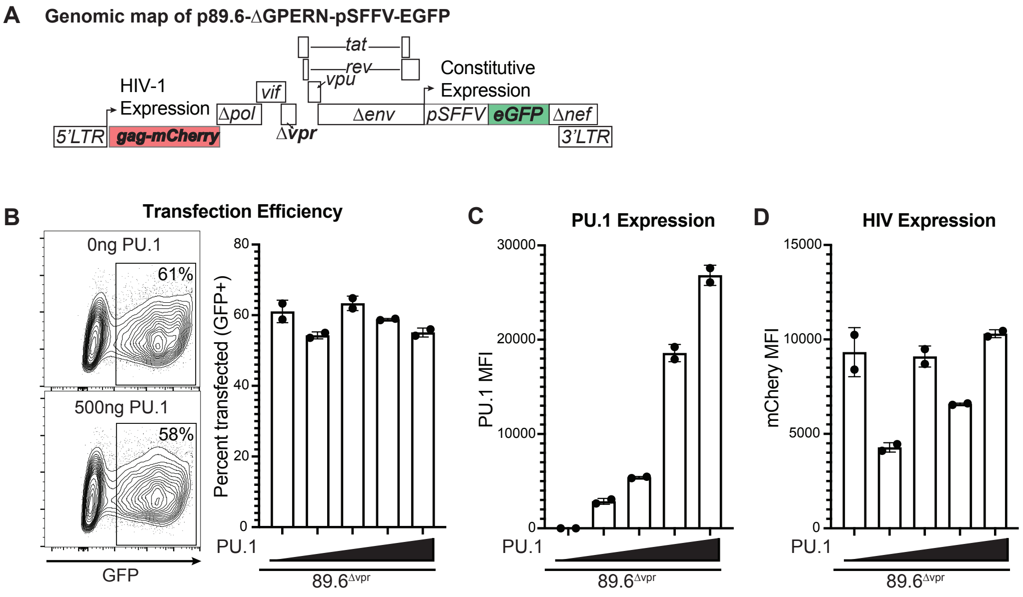

Supplementary Figure 4. PU.1 does not alter HIV-LTR activity. (A) Genomic map for 89.6-derived HIV-1 fluorescent reporter virus (89.6-ΔGPERN-pSFFV-EGFP). (B) Representative flow plots and bar graph assessing transfection rate via GFP expression in HEK 293T cells transfected with the indicated amount of PU.1 construct from Figure 2E plus 500ng of 89.6-ΔGPERN-pSFFV-EGFP from (A). (C) Summary graph of PU.1 expression in GFP+ cells from (B). PU.1 levels were assessed using intracellular staining as described in Methods and measured by flow cytometry. (D) Summary graph of HIV-LTR activity as assessed by mCherry MFI from the same cells as in (C), measured flow cytometrically. All conditions were transfected with 500ng of HIV expression plasmid plus 0, 1, 10, 100, or 500ng of PU.1 plasmid. MFI = mean fluorescence intensity. Source data are provided as a Source Data file.

Figure 4H

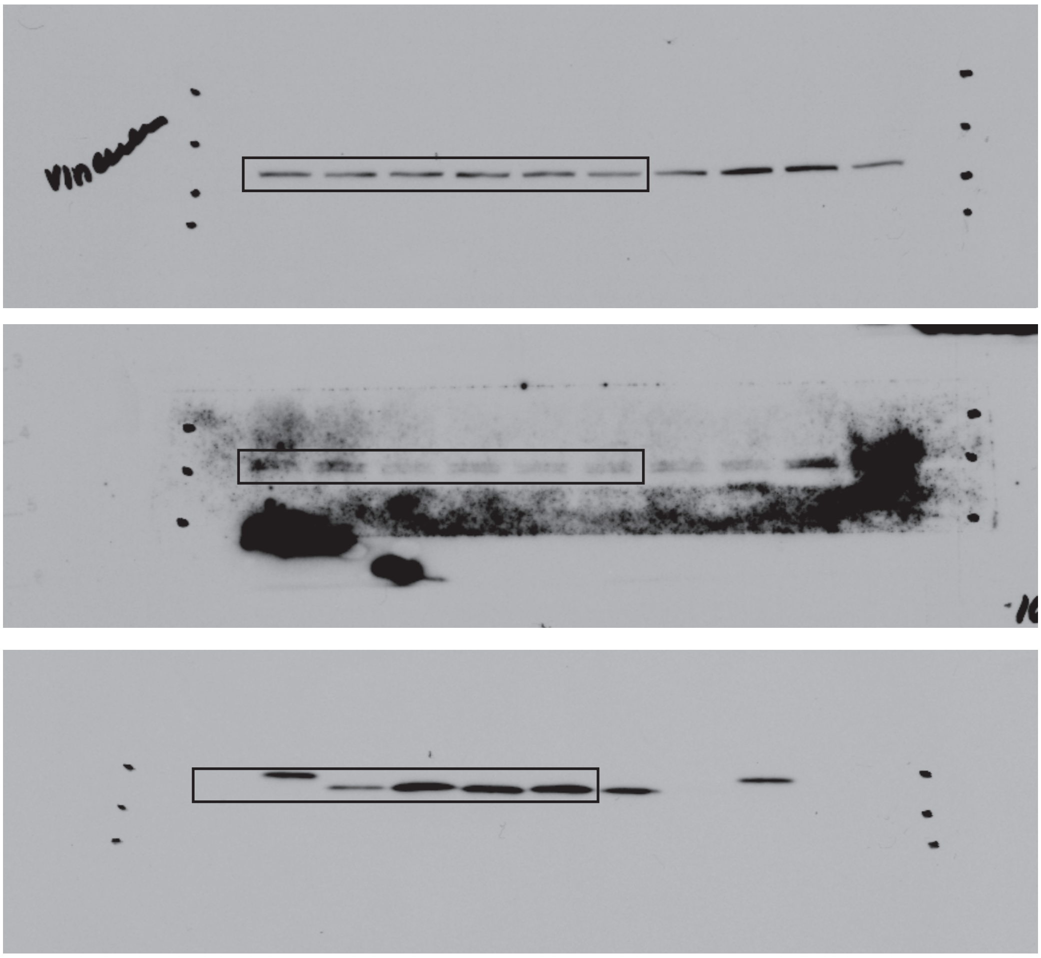

Vinculin

PU.1

FLAG

Figure 6A

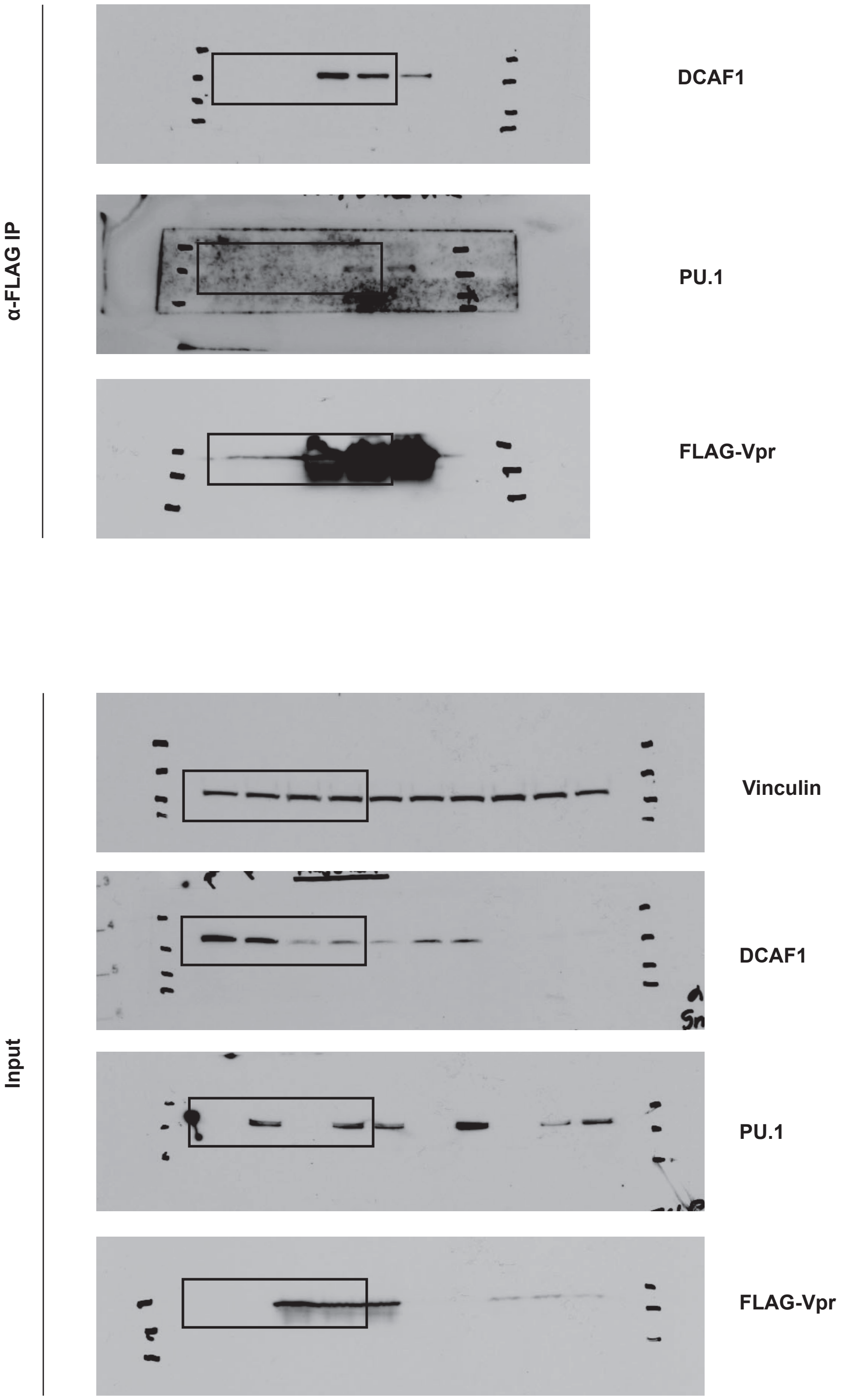

α-FLAG IP
DCAF1
PU.1
FLAG-Vpr
Input
Vinculin
DCAF1
PU.1
FLAG-Vpr

Figure 6B

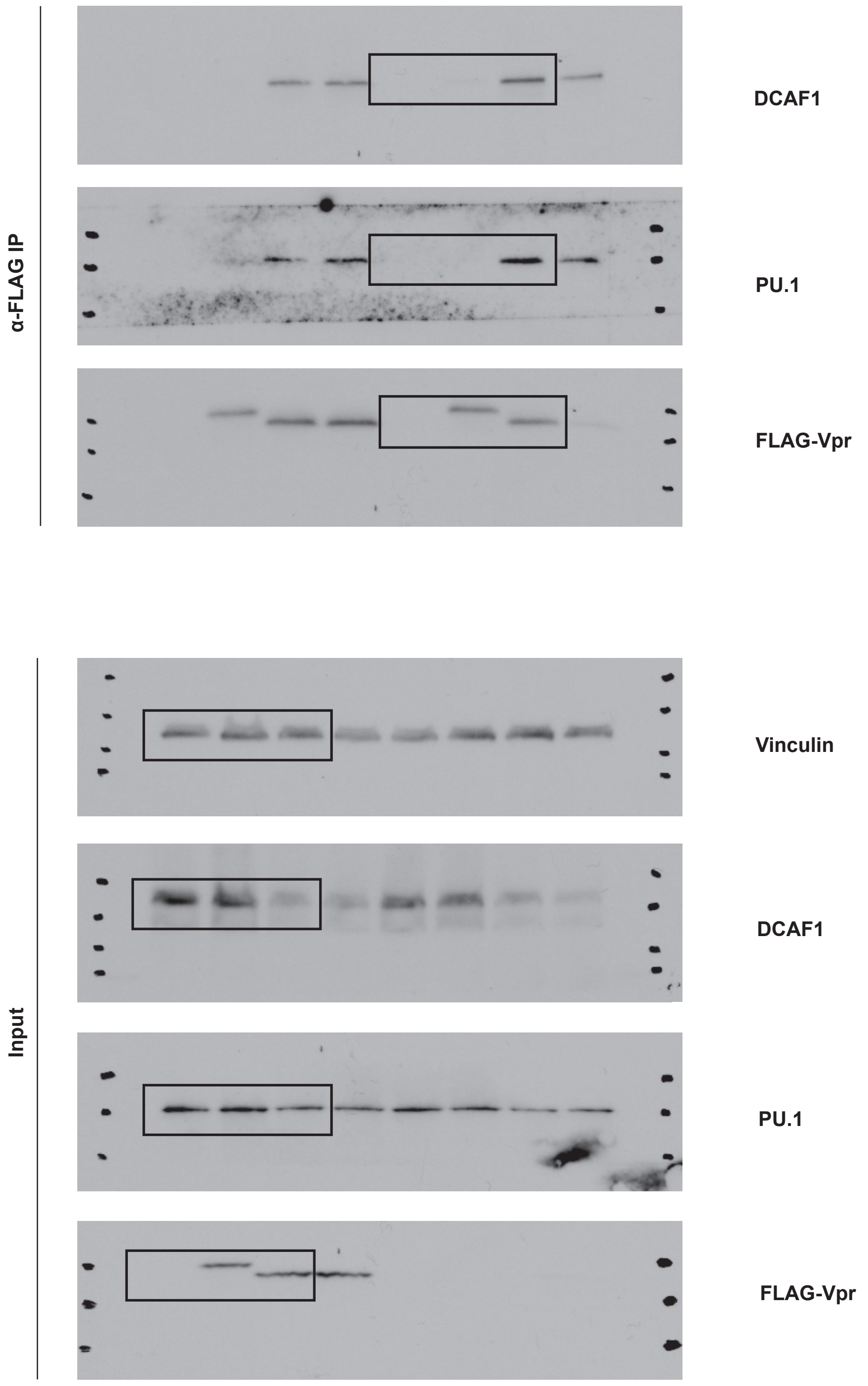
α-FLAG IP
DCAF1
PU.1
FLAG-Vpr
Input
Vinculin
DCAF1
PU.1
FLAG-Vpr

Figure 6C

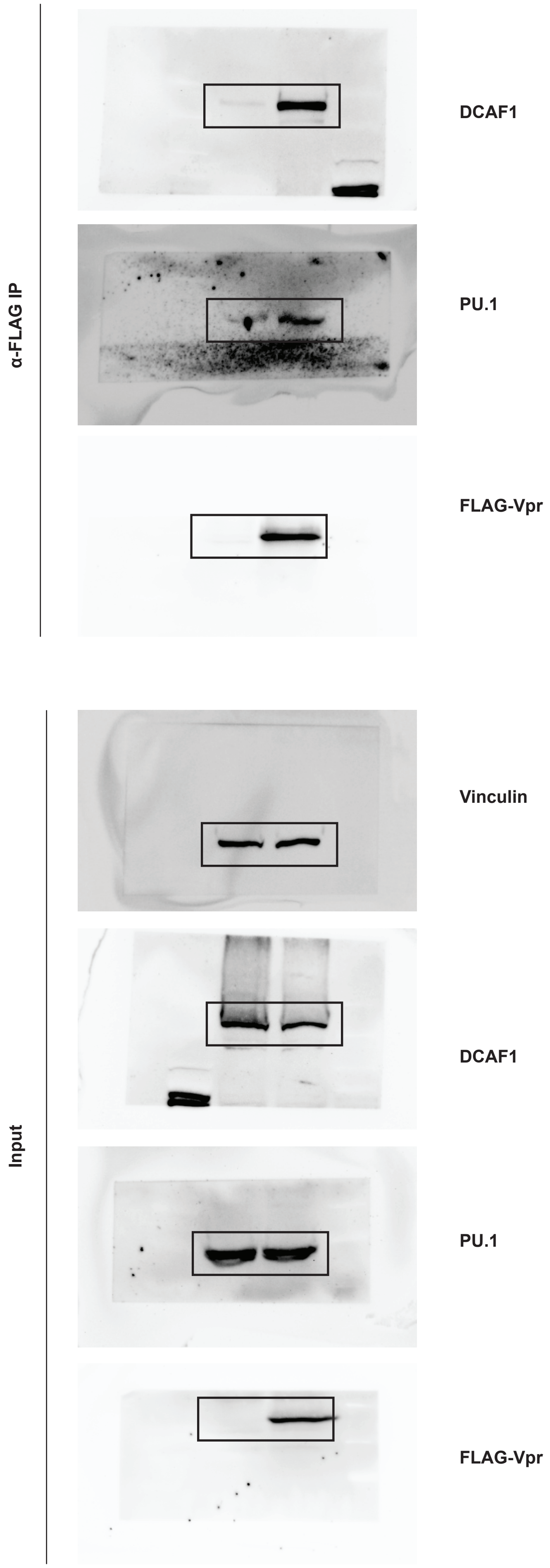
α-FLAG IP
DCAF1
PU.1
FLAG-Vpr
Input
Vinculin
DCAF1
PU.1
FLAG-Vpr

Figure 6D

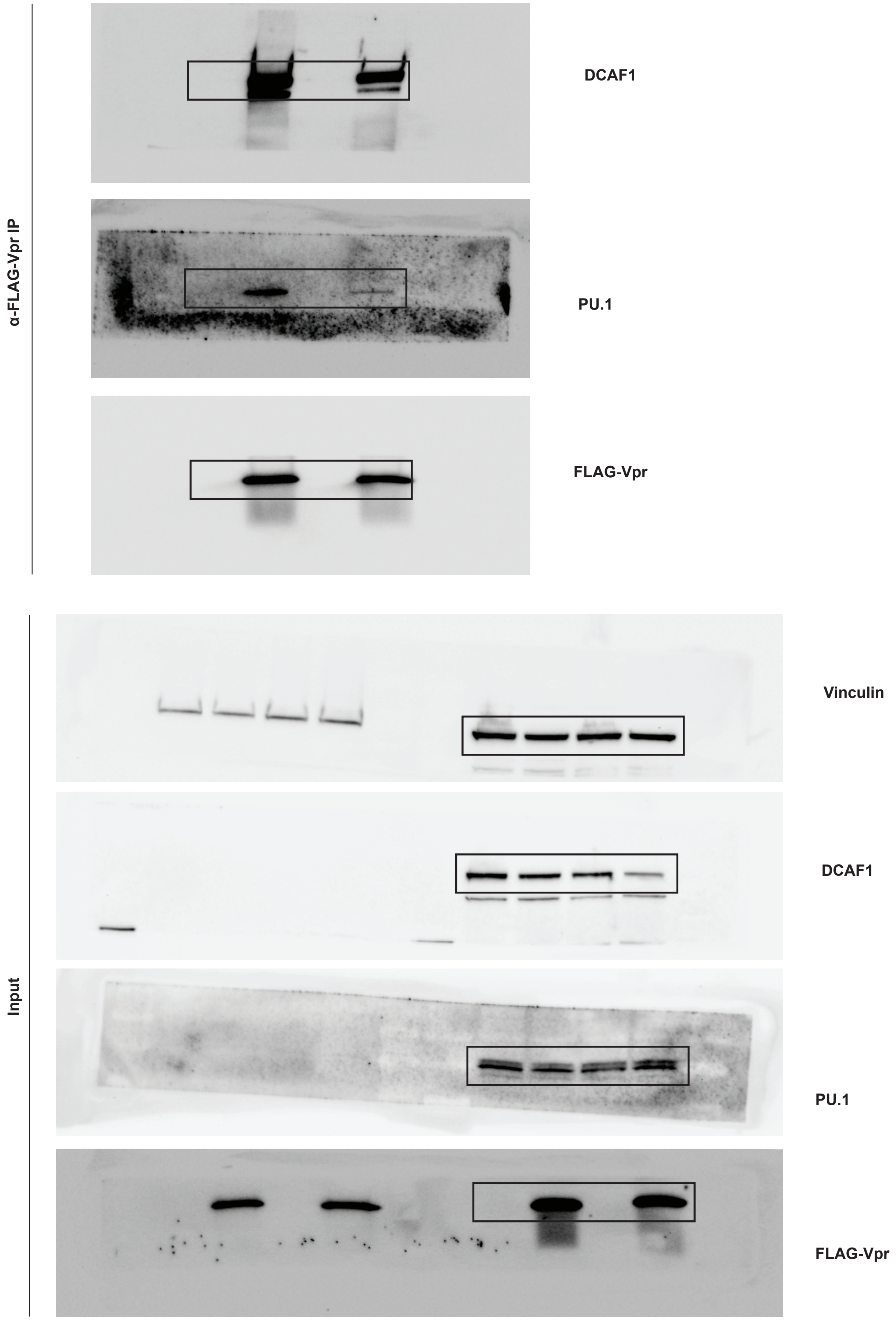

α-FLAG-Vpr IP
DCAF1
PU.1
FLAG-Vpr
Input
Vinculin
DCAF1
PU.1
FLAG-Vpr

Figure 6E

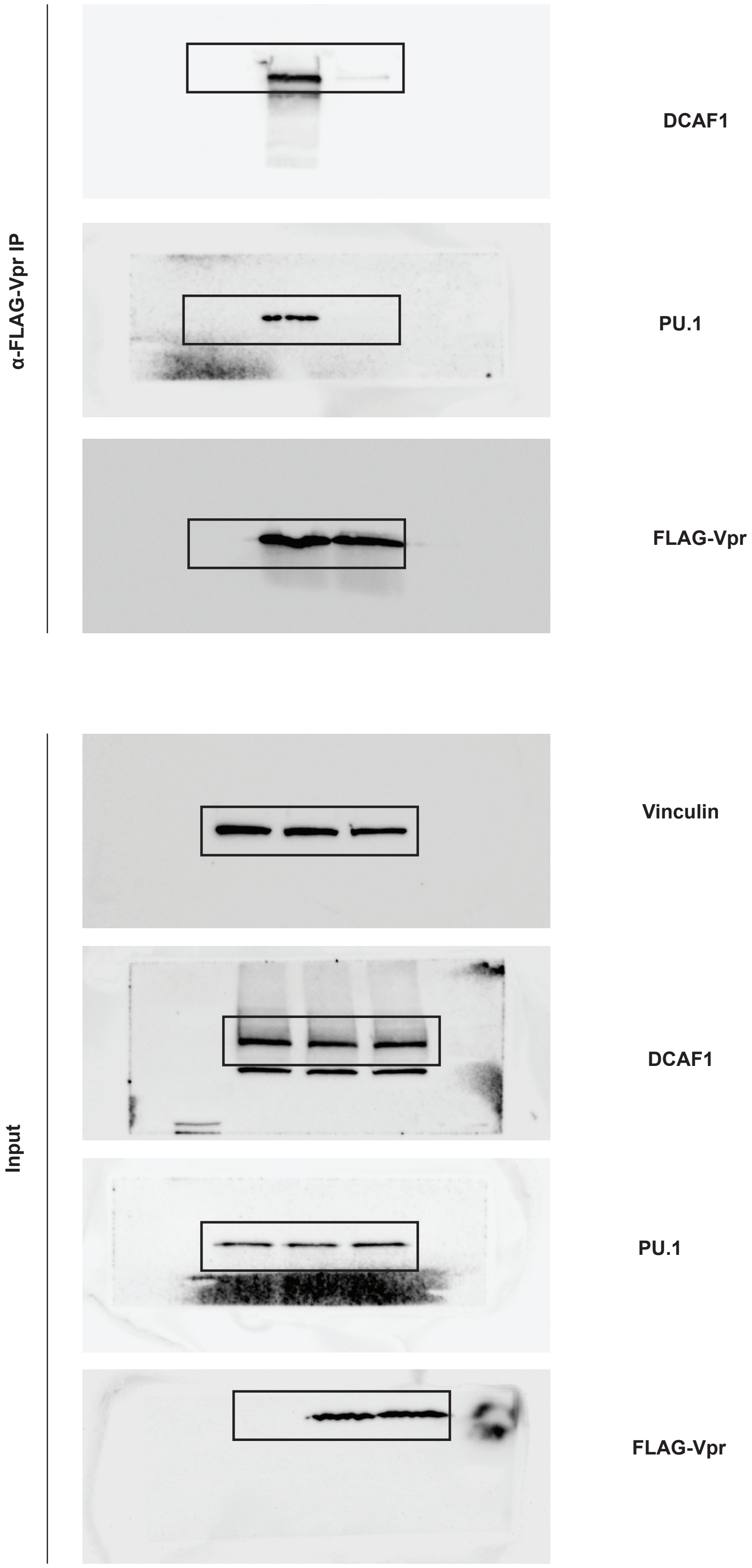
α-FLAG-Vpr IP
DCAF1
PU.1
FLAG-Vpr
Input
Vinculin
DCAF1
PU.1
FLAG-Vpr

Figure 6F

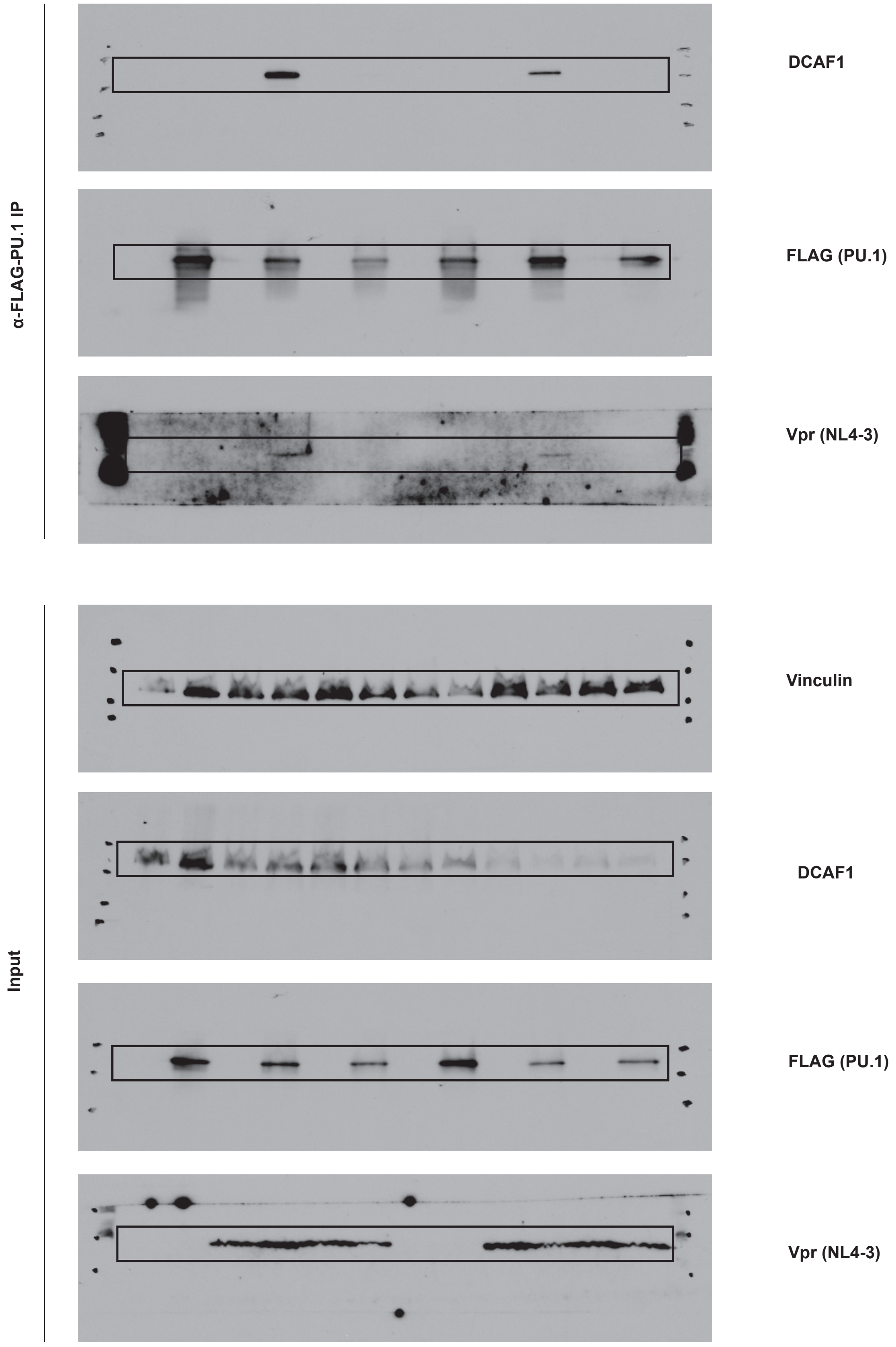
α-FLAG-PU.1 IP
DCAF1
FLAG (PU.1)
Vpr (NL4-3)
Input
Vinculin
DCAF1
FLAG (PU.1)
Vpr (NL4-3)

Figure 6G

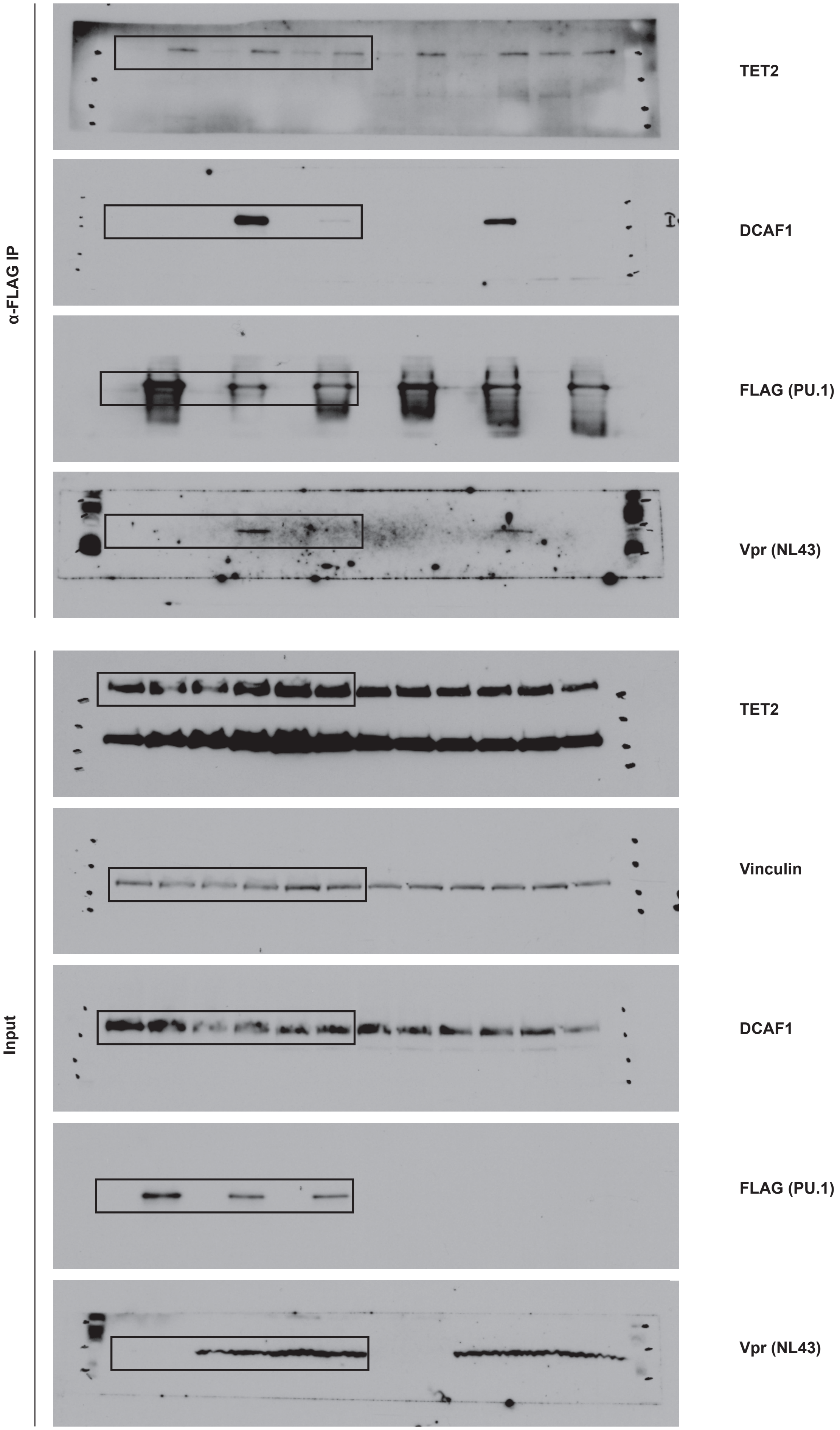
α-FLAG IP
TET2
DCAF1
FLAG (PU.1)
Vpr (NL43)
Input
TET2
Vinculin
DCAF1
FLAG (PU.1)
Vpr (NL43)

Figure 7A

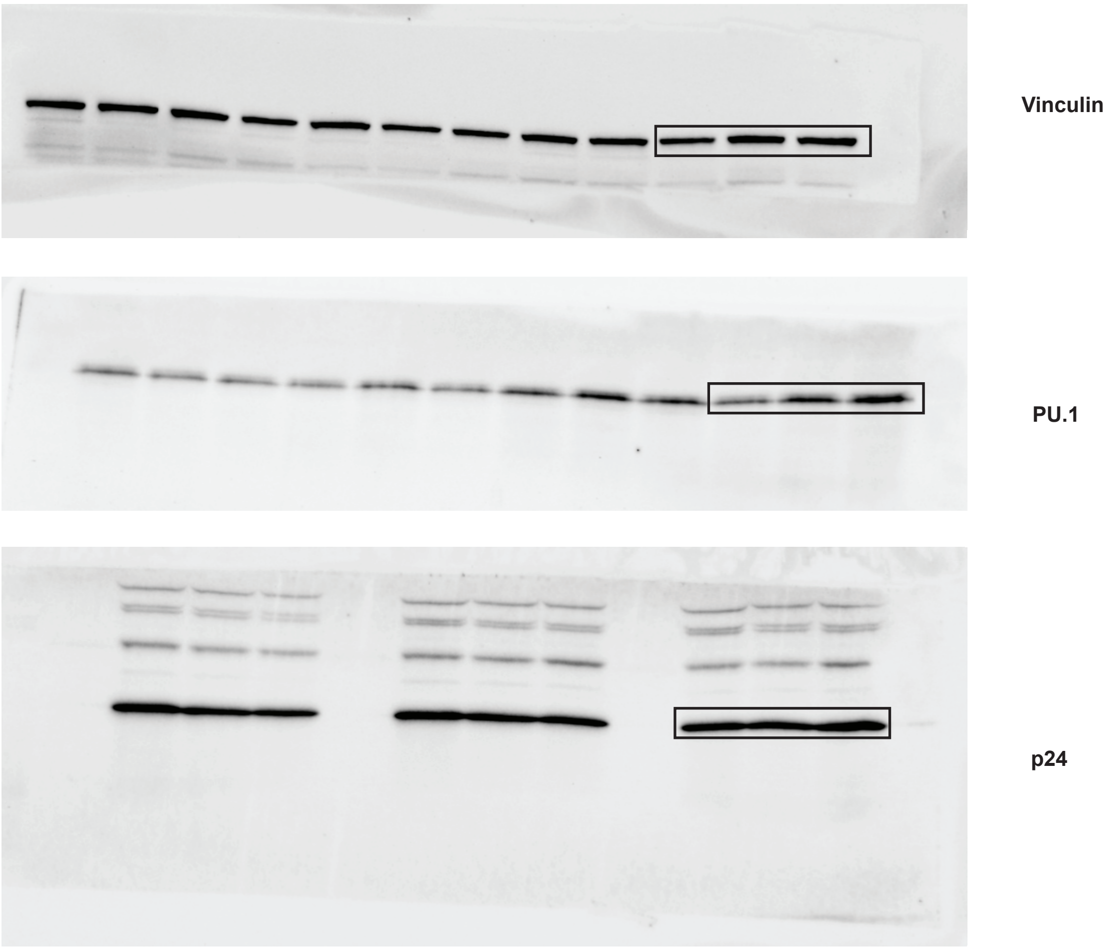

Figure 7C

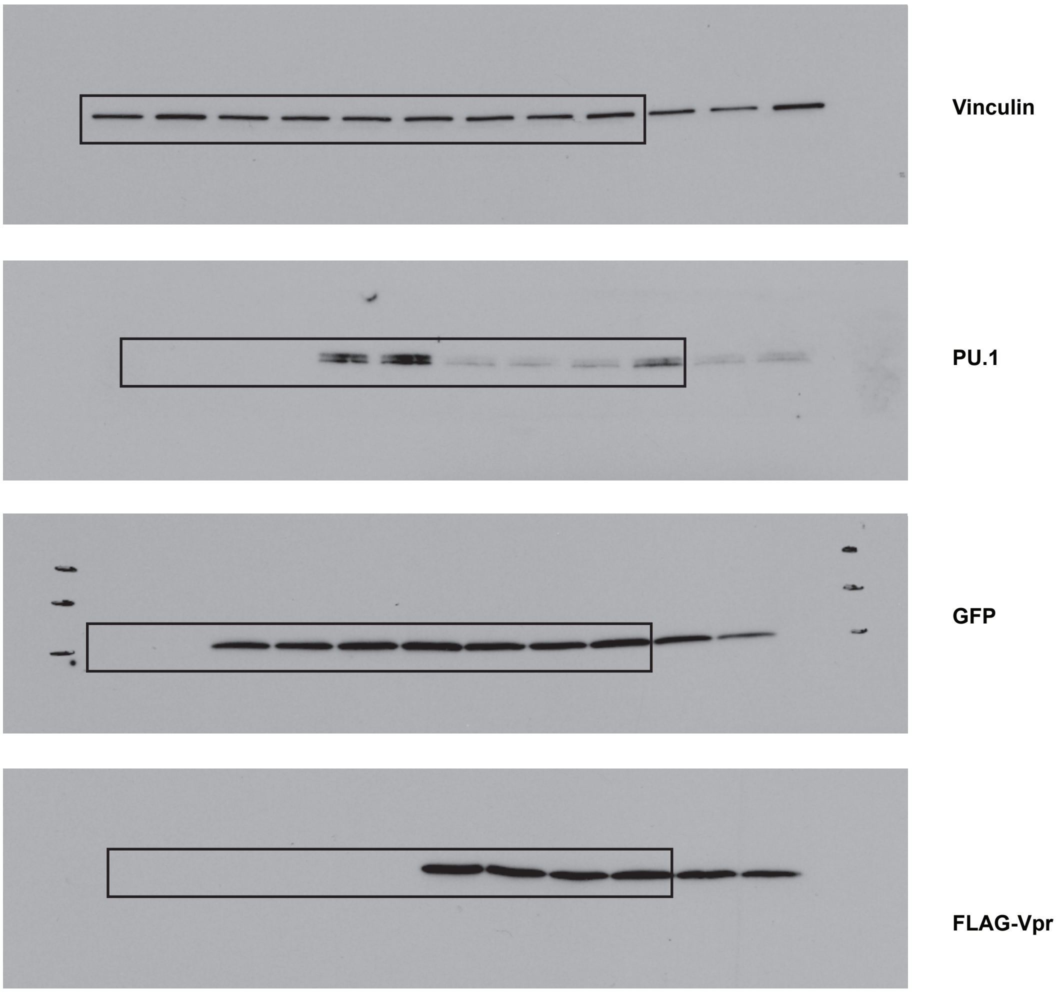

Figure 7D

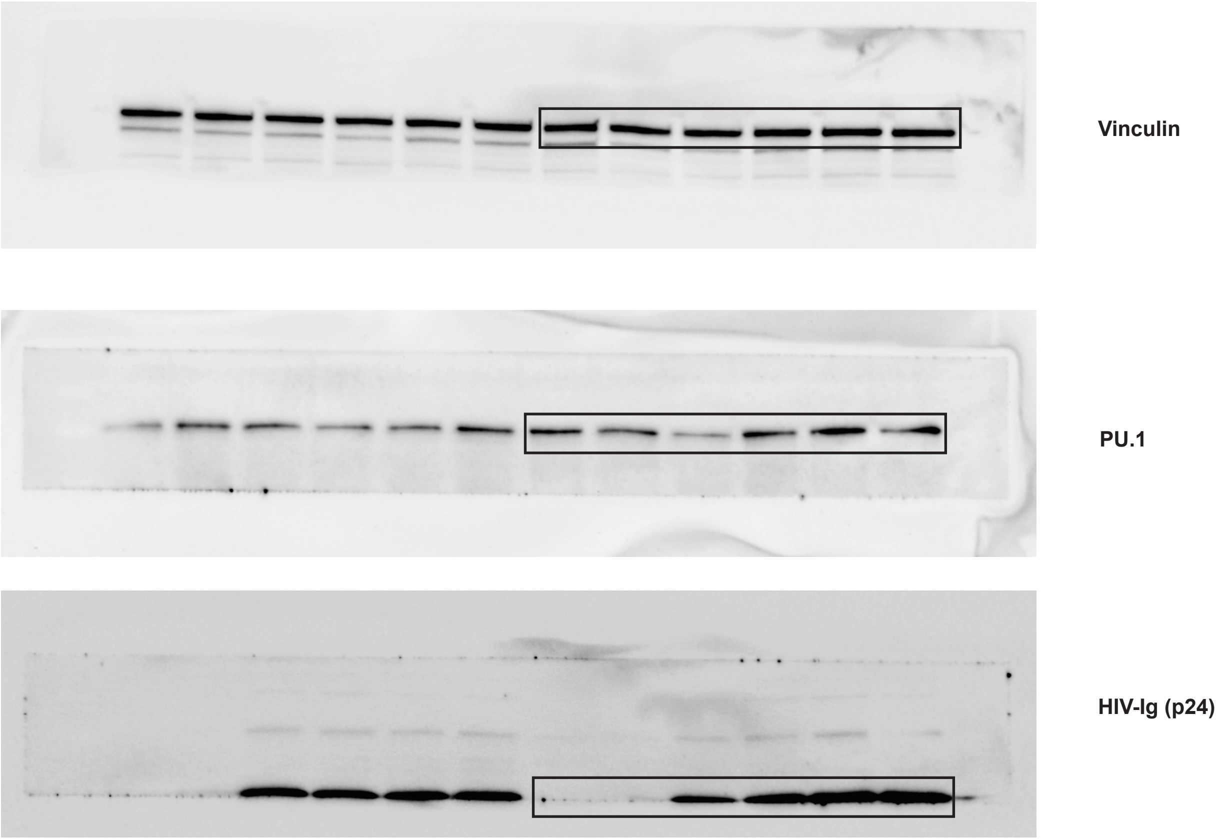

Figure 8B

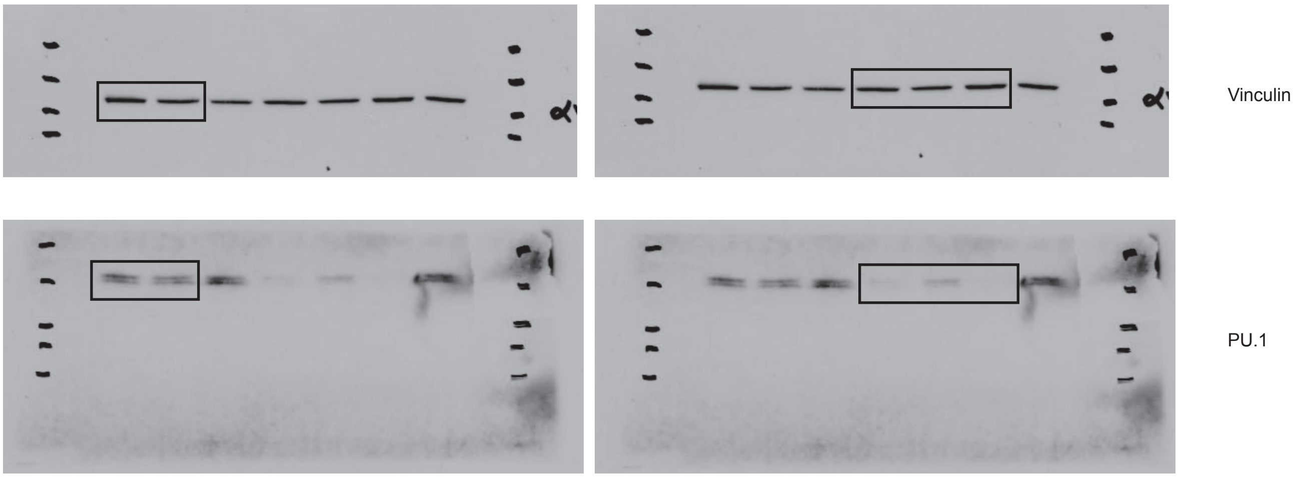

Figure 8C Donor 1

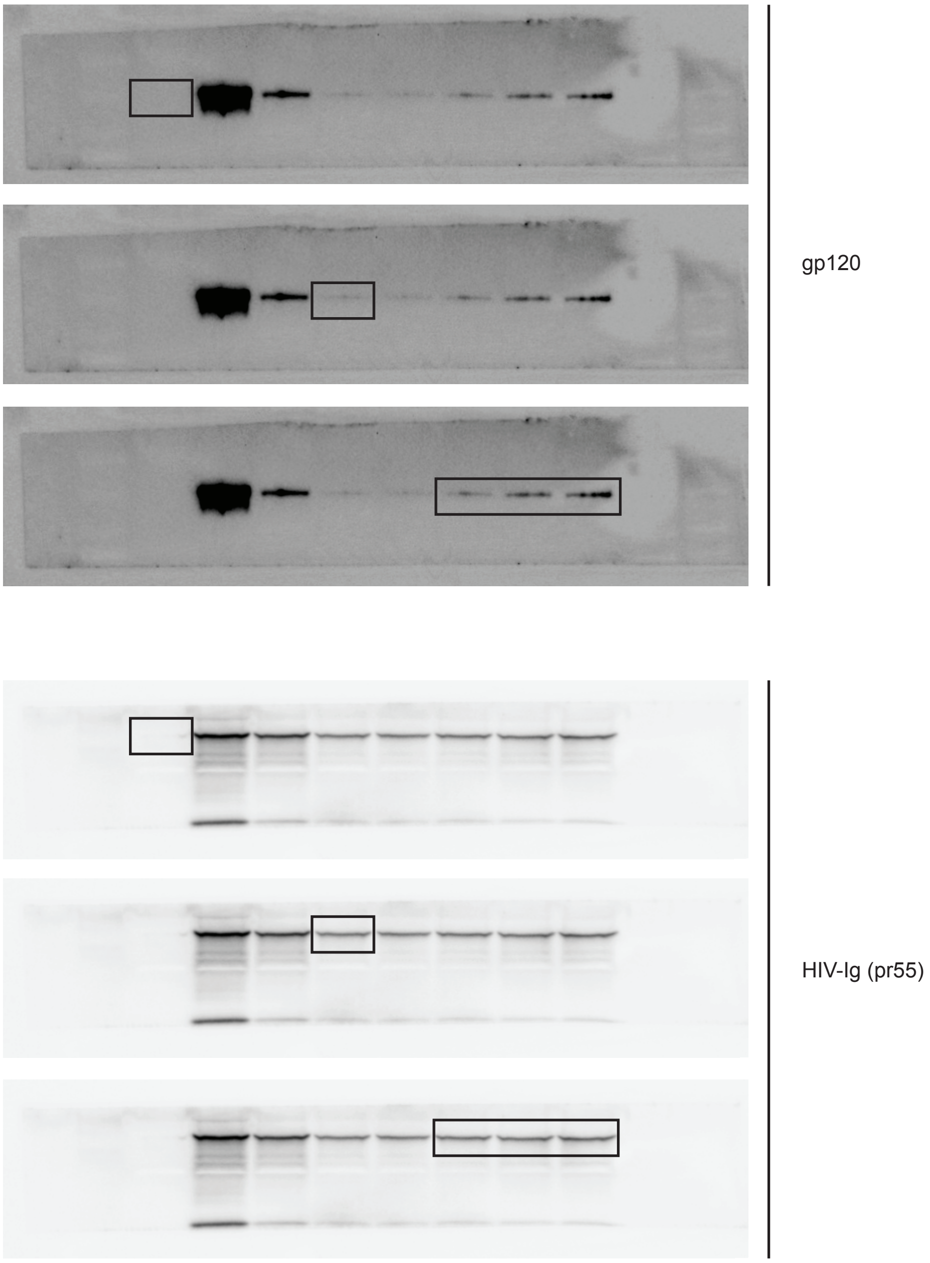

Figure 8C Donor2

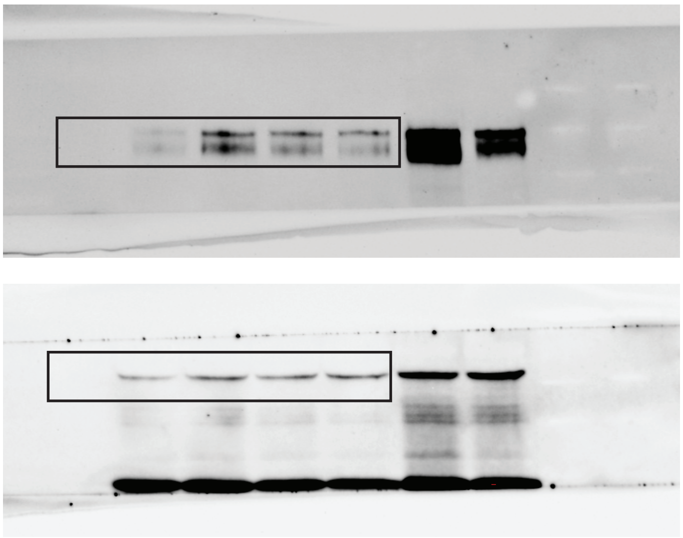

gp120

HIV-Ig (pr55)

Supplementary Figure 2B

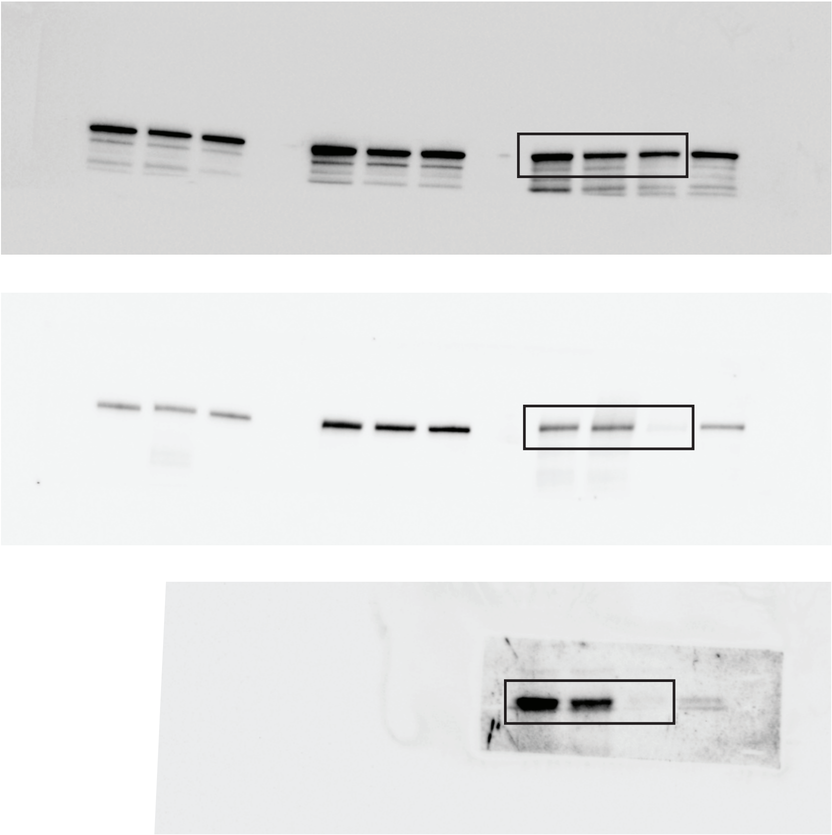

Vinculin

MR

PU.1
