# Supplementary material for: HIV-1 Vpr combats the PU.1-driven antiviral response in primary human macrophages
Source: Nat Commun. 2024 Jun 29;15:5514. doi: 10.1038/s41467-024-49635-w (PMC11217462; doi:10.1038/s41467-024-49635-w)
Supplement: Supplementary file 3 — Reporting Summary [file 41467_2024_49635_MOESM3_ESM.pdf]

nature portfolio

Corresponding author(s): Kathleen L. Collins

Last updated by author(s): Jun 7, 2024

Reporting Summary

Nature Portfolio wishes to improve the reproducibility of the work that we publish. This form provides structure for consistency and transparency in reporting. For further information on Nature Portfolio policies, see our Editorial Policies and the Editorial Policy Checklist.

Statistics

For all statistical analyses, confirm that the following items are present in the figure legend, table legend, main text, or Methods section.

n/a	Confirmed	
☐	☒	The exact sample size (*n*) for each experimental group/condition, given as a discrete number and unit of measurement
☐	☒	A statement on whether measurements were taken from distinct samples or whether the same sample was measured repeatedly
☐	☒	The statistical test(s) used AND whether they are one- or two-sided<br>*Only common tests should be described solely by name; describe more complex techniques in the Methods section.*
☒	☐	A description of all covariates tested
☐	☒	A description of any assumptions or corrections, such as tests of normality and adjustment for multiple comparisons
☐	☒	A full description of the statistical parameters including central tendency (e.g. means) or other basic estimates (e.g. regression coefficient) AND variation (e.g. standard deviation) or associated estimates of uncertainty (e.g. confidence intervals)
☐	☒	For null hypothesis testing, the test statistic (e.g. *F*, *t*, *r*) with confidence intervals, effect sizes, degrees of freedom and *P* value noted<br>*Give P values as exact values whenever suitable.*
☒	☐	For Bayesian analysis, information on the choice of priors and Markov chain Monte Carlo settings
☐	☒	For hierarchical and complex designs, identification of the appropriate level for tests and full reporting of outcomes
☒	☐	Estimates of effect sizes (e.g. Cohen's *d*, Pearson's *r*), indicating how they were calculated

Our web collection on statistics for biologists contains articles on many of the points above.

Software and code

Policy information about availability of computer code

Data collection	Newly generated MDM samples were generated from CellRanger version 3.0.0 (10X Genomics). GFP+ and GFP- HEK 293T cells in the MG132 treatment experiments and for qPCR were sorted on the Sony SH800 cell sorter.
Data analysis	The main functions used for single-cell analysis in this study are open-source and freely available.<br>LIGER at (https://github.com/welch-lab/liger)<br>HOMER at http://homer.ucsd.edu/homer/motif/)<br>GOrilla at (http://cbl-gorilla.cs.technion.ac.il/)<br>REVIGO at (http://revigo.irb.hr/)<br>FIMO at (https://meme-suite.org/meme/doc/fimo.html)<br>Seurat at (https://satijalab.org/seurat/)<br>ImageJ at (https://imagej.net/software/fiji/)<br>FlowJo v10 (https://www.flowjo.com/)<br>GraphPad Prism (https://www.graphpad.com/)

For manuscripts utilizing custom algorithms or software that are central to the research but not yet described in published literature, software must be made available to editors and reviewers. We strongly encourage code deposition in a community repository (e.g. GitHub). See the Nature Portfolio guidelines for submitting code & software for further information.

Data

Policy information about availability of data

All manuscripts must include a data availability statement. This statement should provide the following information, where applicable:

- Accession codes, unique identifiers, or web links for publicly available datasets
- A description of any restrictions on data availability
- For clinical datasets or third party data, please ensure that the statement adheres to our policy

CellRanger version 3.0.0 processed data generated in the manuscript have been deposited in GEO under accession code GSE220574 [https://www.ncbi.nlm.nih.gov/geo/query/acc.cgi?acc=GSE220574]. Raw sequencing data have been deposited in NCBI dbGAP database under accession code phs002915.v2.p1. The raw data are available under restricted access due to data privacy concerns and can be obtained by requesting access from NCBI. TF motif data and analysis from HOMER (http://homer.ucsd.edu/homer/) are described in the Methods section. Source data are provided as a Source Data file.

Research involving human participants, their data, or biological material

Policy information about studies with human participants or human data. See also policy information about sex, gender (identity/presentation), and sexual orientation and race, ethnicity and racism.

Reporting on sex and gender	No sex or gender based information was provided and no sex or gender based analyses were performed.
Reporting on race, ethnicity, or other socially relevant groupings	No race, ethnicity, or other socially relevant information was collected or considered in this study.
Population characteristics	None collected.
Recruitment	Monocytes were isolated from anonymous donors by apheresis and obtained from the New York Blood Center Component Laboratory
Ethics oversight	The use of human blood from anonymous, de-identified donors was classified as non-human subject research in accordance with federal regulations and therefore not subject to formal IRB review.

Note that full information on the approval of the study protocol must also be provided in the manuscript.

Field-specific reporting

Please select the one below that is the best fit for your research. If you are not sure, read the appropriate sections before making your selection.

☒ Life sciences ☐ Behavioural & social sciences ☐ Ecological, evolutionary & environmental sciences

For a reference copy of the document with all sections, see nature.com/documents/nr-reporting-summary-flat.pdf

Life sciences study design

All studies must disclose on these points even when the disclosure is negative.

Sample size	The number of cells for the MDM samples were determined based on 10X Genomics protocols.
Data exclusions	When preprocessing each MDM single-cell sample, cell and gene filtering based on expression was performed. All raw data are made public.
Replication	All findings discussed in the manuscript are reproducible in a minimum of two independent donors or replicates.
Randomization	There were no interventions to randomize in this study.
Blinding	Blinding is not relevant to our study.

Reporting for specific materials, systems and methods

We require information from authors about some types of materials, experimental systems and methods used in many studies. Here, indicate whether each material, system or method listed is relevant to your study. If you are not sure if a list item applies to your research, read the appropriate section before selecting a response.

Materials & experimental systems

n/a	Involved in the study
☐	☒ Antibodies
☐	☒ Eukaryotic cell lines
☒	☐ Palaeontology and archaeology
☒	☐ Animals and other organisms
☒	☐ Clinical data
☒	☐ Dual use research of concern
☒	☐ Plants

Methods

n/a	Involved in the study
☒	☐ ChIP-seq
☐	☒ Flow cytometry
☒	☐ MRI-based neuroimaging

Antibodies

Antibodies used

Antibodies to Vinculin (1:1000, cat# V9131, Millipore Sigma), DCAF1 (1:1000, cat# 11612-1AP, Proteintech), PU.1 (1:100, cat# 2266S, Cell Signalling Technology), FLAG (1:1000, cat# F1804, Millipore Sigma), TET2 (1:250, cat#MABE462, EMD Millipore), Vpr (1:500, AIDS Reagent Program cat# 3951 from Dr. Jeffrey Kopp), pr55 and p24 (1:1000, AIDS Reagent Program cat# 3957), gp120 (1:1000, AIDS Reagent Program cat# 288), GFP (1:1000, cat# ab13970, Abcam) were used for immunoblot analysis. Secondary HRP conjugated antibodies against murine (1:10000, rat anti-mouse IgG1, eBioscience), rabbit (1:5000, goat anti-rabbit IgG, Invitrogen), sheep (1:20000, rabbit anti-sheep IgG, Dako), and human (1:10000, goat anti-human IgG, ThermoFisher) were also used. Antibodies to PU.1 (1:100, clone 7C6B05, BioLegend), FLAG (1:3000, cat# 637324, BioLegend) were used for flow cytometry. Antibodies to ISG15 (1:450, cat# 15981-1-AP, Proteintech), IFITM3 (1:400, cat# 11714-1-AP, Proteintech), and AlexaFluor 647 (1:200, A21244, Fisher Scientific) secondary antibody were used for immunofluorescence. CAp24 (1:400, clone KC57-PE cat# 6604667, Beckman Coulter) was used for both flow cytometry and immunofluorescence.

Validation

Each antibody was validated by immunofluorescent staining by flow cytometric analysis with positive and negative controls to ensure specificity.

Eukaryotic cell lines

Policy information about cell lines and Sex and Gender in Research

Cell line source(s)

K562 and HEK 293T from ATCC

Authentication

All cell lines were independently authenticated using STR profiling.

Mycoplasma contamination

All cell lines tested negative for mycoplasma.

Commonly misidentified lines (See ICLAC register)

No commonly misidentified cell lines were used in this study.

Plants

Seed stocks

Report on the source of all seed stocks or other plant material used. If applicable, state the seed stock centre and catalogue number. If plant specimens were collected from the field, describe the collection location, date and sampling procedures.

Novel plant genotypes

Describe the methods by which all novel plant genotypes were produced. This includes those generated by transgenic approaches, gene editing, chemical/radiation-based mutagenesis and hybridization. For transgenic lines, describe the transformation method, the number of independent lines analyzed and the generation upon which experiments were performed. For gene-edited lines, describe the editor used, the endogenous sequence targeted for editing, the targeting guide RNA sequence (if applicable) and how the editor was applied.

Authentication

Describe any authentication procedures for each seed stock used or novel genotype generated. Describe any experiments used to assess the effect of a mutation and, where applicable, how potential secondary effects (e.g. second site T-DNA insertions, mosiacism, off-target gene editing) were examined.

Flow Cytometry

Plots

Confirm that:

☒ The axis labels state the marker and fluorochrome used (e.g. CD4-FITC).

☒ The axis scales are clearly visible. Include numbers along axes only for bottom left plot of group (a 'group' is an analysis of identical markers).

☒ All plots are contour plots with outliers or pseudocolor plots.

☒ A numerical value for number of cells or percentage (with statistics) is provided.

Methodology

Sample preparation	For cells requiring intracellular staining using antibodies directed against HIV Gag p24, FLAG-Vpx and -Vpr, and PU.1, paraformaldehyde-fixed cells were permeabilized with 0.1% Triton-X in PBS for 2min followed by incubation with antibody for 30min at room temperature.
Instrument	Cytek Aurora
Software	All flow cytometry data was analyzed using FlowJo software (TreeStar, Ashland, Oregon).
Cell population abundance	Post-sort purity for GFP positivity was not measured in this study, but the sorting by GFP+ cells was confirmed during western blotting for GFP protein in the sorted samples.
Gating strategy	In all experiments, cells were gated sequentially by forward scatter vs. side scatter for cells and then by forward scatter area vs. height to exclude doublets. The gating strategy is shown in Supplemental Figure 3E.

☒ Tick this box to confirm that a figure exemplifying the gating strategy is provided in the Supplementary Information.
